# Comparative Life Cycle Assessment of Bacterial and Thermochemical Retting of Hemp

**DOI:** 10.3390/ma17164164

**Published:** 2024-08-22

**Authors:** Yu Fu, Hongmei Gu, H. Felix Wu, Sheldon Q. Shi

**Affiliations:** 1 Department of Mechanical Engineering, University of North Texas Discovery Park, 3940 N Elm St., Denton, TX 76207, USA; yufu@my.unt.edu; 2 USDA Forest Service, Forest Products Laboratory, One Gifford Pinchot Drive, Madison, WI 53726, USA; hongmei.gu@usda.gov; 3 Office of Energy Efficiency and Renewable Energy, U.S. Department of Energy, Washington, DC 20585, USA; felix.wu@ee.doe.gov

**Keywords:** hemp bast fiber, bacterial retting, life cycle assessment, environmental impact, cumulative energy demand, plant-based materials

## Abstract

The processes of hemp bast fiber retting, forming, and drying offer the opportunity for value-added products such as natural fiber-reinforced composites. A new process for the retting of raw bast fibers through enzyme-triggered self-cultured bacterial retting was developed in the lab-scale setup. This study focused on comparing the energy consumption and environmental impacts of this bacterial retting process with the thermochemical retting process currently widely used to obtain lignocellulosic fibers for composites. The gate-to-gate life cycle assessment (LCA) models of the two retting processes were constructed to run a comparison analysis using the TRACI (the tool for the reduction and assessment of chemical and other environmental impacts) method for environmental impacts and the cumulative energy demand (CED) method for energy consumptions. This work has demonstrated the advantages of the bacterial retting method from an environmental standpoint. The result of our research shows about a 24% gate-to-gate reduction in CED for bacterial retting and 20–25% lower environmental impacts relating to global warming, smog formation, acidification, carcinogenics, non-carcinogenics, respiratory effects, ecotoxicity, and fossil fuel depletion when compared to that of thermochemical retting.

## 1. Introduction

Renewable natural fibers are gaining popularity due to the environmental protection challenges associated with fossil fuel-based fibers. The automotive industry has been making efforts to replace traditional glass- or carbon fiber-reinforced polymer composites with natural fiber composites for some automotive parts. Because natural fiber composites are lightweight, fuel efficiency or battery mileage can be reduced, thus reducing greenhouse gas (GHG) emissions. A 10% reduction in vehicle weight could potentially yield a fuel economy improvement of 6–8% [1]. It has been demonstrated that the use of natural fiber composites in the construction and building industry can reduce the carbon footprint [2]. Hemp-based boards showed lower GHG emissions compared to gypsum plasterboards due to the sequestered biogenic carbon during hemp growth [3]. Galan-Marin et al. studied the global warming potential (GWP) of natural fiber composites in construction [4]. Natural fiber composite block walls (containing natural fiber wool and natural polymer calcium alginate) have a lower total GWP than that of the fired clay brick walls and concrete block walls. Other studies also showed that the environmental impact of the buildings could be decreased by using bio-based materials [5,6]. Among the many natural fibers, hemp has received increasing attention in the United States after it was fully legalized for growing in 2018 [7].

Retting is a process to convert the harvested biomass into fibers. Proper retting ensures the quality of the hemp bast fibers for fiber-reinforced polymer composites. Zimniewska [8], Manian et al. [9], and Lee et al. [10] summarized the different retting methods and their advantages and disadvantages. With the study of microorganisms in the retting process, biological retting methods, including enzymatic retting, bacterial retting, and fungal retting, have become increasingly popular in recent years. The contribution of bacteria in the retting process is being explored. Zhao et al. found that the bacteria Bacillus cereus HDYM-02 significantly changed bacterial successions during flax retting and accelerated the process compared to natural retting [11]. The cellulase-free crude enzyme produced by Bacillus cereus HDYM-02 contained high pectinase and mannanase activity, which acted synergistically in the retting of flax [12]. It has been proven that pectinolytic bacteria reduced the jute retting period and improved fiber quality [13]. Although many retting methods have been practiced in labs, studies on the environmental impacts of these retting methods are limited.

Life cycle assessment (LCA) is a holistic method to systematically assess the environmental impact of products, processes, or services from cradle-to-grave, cradle-to-gate, or gate-to-gate system boundaries, following the ISO standards [14,15]. LCA has been applied to evaluate natural fiber-reinforced products such as kenaf fiber-reinforced composites [16,17,18], flax fiber-reinforced composites [19], and hemp fiber-reinforced composites [20]. Generally, the interfacial bonding between fibers and the matrix [21] plays a pivotal role in regulating the properties and performance of composites [22]. However, little is known about the environmental impact of fiber retting. Corona and Birved assessed the life cycle performance for enzymatic retting and field retting of hemp [23]. It was found that the enzymatic retting process had a higher environmental impact than the field retting process. The enzymatic retting scenario in this study included hydrothermal pretreatment and enzymatic (endo-polygalacturonase and pectin-lyase) treatment with a NaOH buffer. The increase in the environmental impact for this enzymatic retting scenario was attributed to the consumption of electricity and auxiliary material. Considering the final application of the composite material in car doors instead of interior furniture, the environmental impact of enzymatic retting was reduced [23]. In addition to obtaining the overall environmental impact of natural fiber-reinforced composites, it is necessary to understand the environmental impact of emerging retting methods in order to find alternative ways that may further reduce the impact to the environment. In this study, a comparative LCA of thermochemical retting and enzyme-triggered self-cultured bacterial retting was conducted using SimaPro v9.1 software with the DATASMART Life Cycle Inventory (LCI) database Package (https://simapro.com/products/datasmart-lci-package/ accessed on 27 October 2022).

## 2. Methods and Materials

### 2.1. Goal and Scope

This study aimed to assess the environmental impacts associated with a laboratory scale enzyme-triggered self-cultured bacterial retting process and compare those impacts with the traditional thermochemical retting process. A functional unit is defined to provide a reference point for quantification and comparison of the two processes’ environmental impacts. The functional unit for this study is defined as 1 g dry mass of retted hemp fiber with comparable properties that are ready to be delivered to the composite manufacturing “factory gate”. The materials and energy were appropriately scaled up to produce this 1 g hemp fiber.

### 2.2. System Boundary

**Figure 1:**
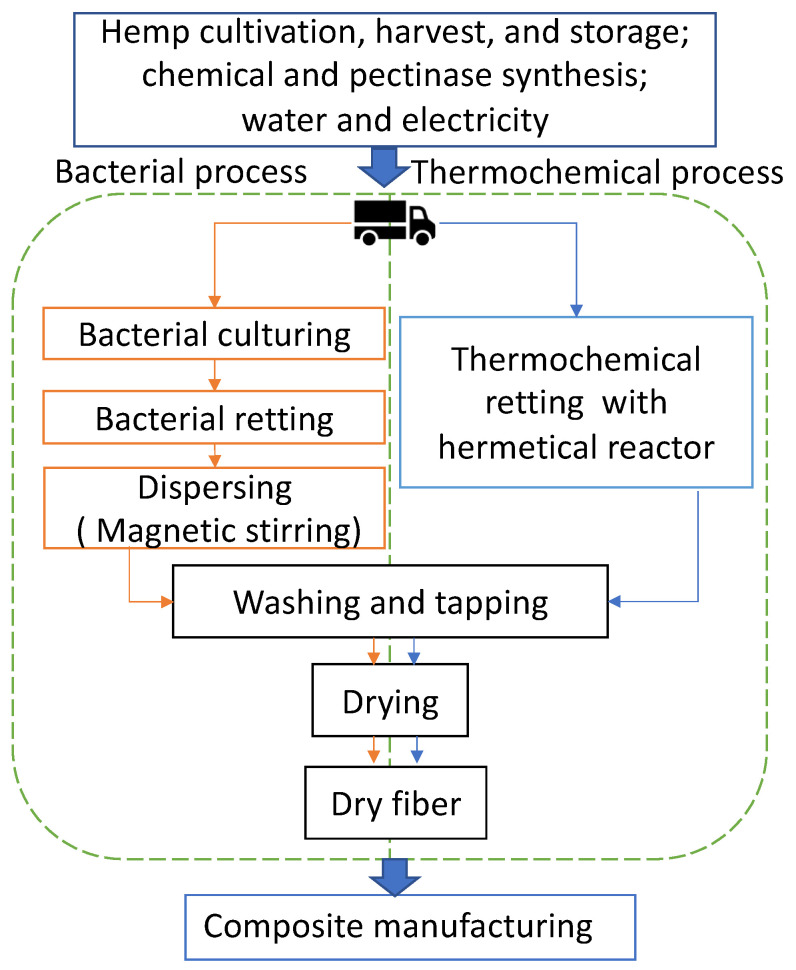
Flow chart of the processes of lab-scale bacterial and thermochemical fiber treatments. Green dotted lines define the system boundary of the comparative LCA.

The system boundary of this comparative LCA study is gate-to-gate from the raw hemp fiber shipping to the lab for fiber-retting treatment ready to be shipped out for further manufacturing into composites. Figure 1 shows the process and boundary system. The comparison included transportation of raw hemp fiber from distributors to the lab, fiber retting, and mat forming until the final product fiber mat is ready to be delivered to the composite manufacturing “factory gate” of both processes. The system input in this study included hemp bast fiber, energy (in the form of electricity), water, chemical or pectinase, and raw material transportation from distributor to lab. Since very little information is available on the environmental impact of pectinase production, data on an established enzyme (alpha-amylase, Novozymes Liquozyme^®^) were researched and used in the LCA modeling of the bacterial process. All the transportation of raw materials (hemp bast fibers, NaOH pellet, and pectinase) from distributors to the lab were included. Manufacturing of process equipment was excluded because laboratory-scale equipment is used for many processes whose contribution is considered negligible over the life of the equipment [24]. The corresponding outputs are the final product hemp fiber, wastewater from the retting process, and emissions associated with upstream raw material processing and energy production.

### 2.3. Description of Processes

Fiber mats manufactured from the bacterial retting and fabrication processes included three stages: (1) bacterial aggregation triggered by pectinase, (2) bacterial retting, and (3) mat production (Figure 1). First, short bast fiber (20 g, 0.25 in) was immersed in pectinase solution (1000 mL, 1% *w*/*v*) and incubated in 40 °C water to trigger the aggregation of bacteria. Subsequently, the fibers were filtered out and the remaining liquid portion was used to ret the next batch of fibers (20 g, 0.25 in) in the 40 °C water bath for three days. Finally, the retted fibers were dispersed in water for 3 h under magnetic stirring (500 rpm), followed by washing and tapping in water with a mesh mold to form 4-inch by 6.5-inch mats. The total water consumption for this stage was 1000 mL. Before they were ready for the composite manufacturing gate, the wet fiber mats were dried in an oven at 80 °C for 10 h.

The conventional chemical retting process was the one-hour alkali (NaOH, 5%, *w*/*v*) retting process in a hermetical reactor (251 M, Parr Instrument Co., Moline, IL, USA) at 160 °C [25]. Unlike bacterial retting, fibers retted by this process need to be washed several times. The total water consumption was 3000 mL to obtain clear fiber from raw hemp bast fiber (20 g). The tapping and drying of wet mats were the same as the bacterial process.

### 2.4. Life Cycle Inventory and Life Cycle Inventory Analysis

Table 1 lists the materials and energy inputs to produce this 1 g hemp fiber. The life cycle inventory data of each stage were quantified, and the impacts were modeled in SimaPro v9.1 software. Ozone depletion (kg CFC-11 eq), global warming (kg CO_2_ eq), smog formation (kg O_3_ eq), acidification (kg SO_2_ eq), eutrophication (kg N eq), carcinogenics (CTUh), non-carcinogenics (CTUh), respiratory effects (kg PM2.5 eq), ecotoxicity (CTUe), and fossil fuel depletion (MJ surplus) were evaluated using the embedded TRACI 2.1 impact method [26] and are reported in the following section.

### 2.5. FTIR Spectroscopy

For providing chemical characterization of hemp bast before and after retting, FT infrared spectroscopy (FTIR) was carried out on Bruker Invenio R device within the range from 400 to 4000 cm^−1^.

## 3. Results and Discussion

The contributions of six different types of energy were investigated (Table 2). The damage categories are divided into nonrenewable (e.g., fossil, nuclear, and biomass) and renewable (e.g., biomass, wind, solar, geothermal, and water), while the corresponding indicators were calculated in mega joule (MJ). A total cumulative energy demand (CED) of 2.862 MJ/g dry hemp fiber for thermochemical retting was required. Non-renewable fossil was the highest and had a major impact, accounting for approximately 99% of the total CED. Compared to the thermochemical retting process, the cumulative energy demand to produce 1 g of retted hemp fiber through the bacterial retting process saved 23.9% of energy. The impact category of total renewable energy for bacterial retting was −1.6 × 10^−5^ MJ/g retted hemp fiber, which indicated the potential renewable energy saving.

The environmental performance of the two processes is shown in Table 3 for each impact category. The global warming potential for bacterial retting was calculated at 0.132 kg CO_2_ eq/g dry hemp fiber produced. This is around 79% of that for thermochemical retting (0.167 kg CO_2_ eq/g dry hemp fiber produced). The ecotoxicity of the bacterial retting process was 0.099 CTUe, and it is approximately 25% lower than that of the thermochemical retting process (0.132 CTUe).

**Figure 2:**
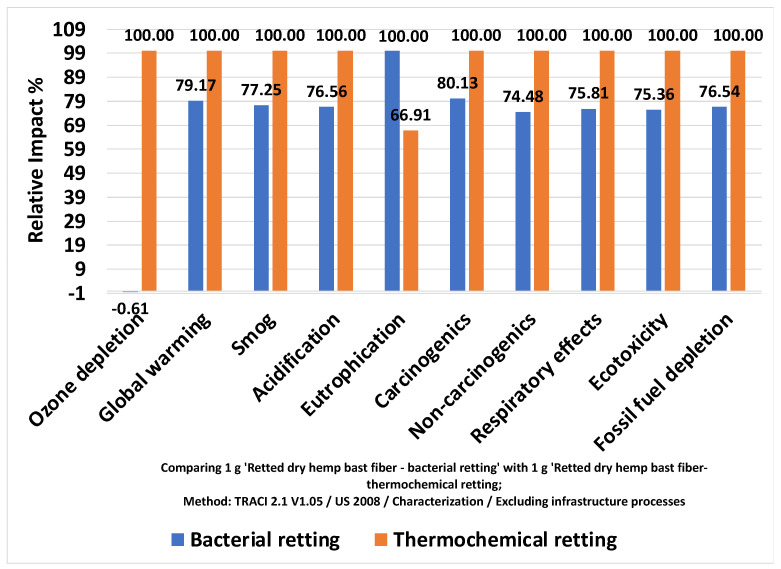
Comparison of environmental impacts for bacterial retting and thermochemical retting.

The gate-to-gate LCA outputs (Figure 2) showed that the bacterial retting process has less environmental impact than that from the thermochemical retting process for all environmental categories except for eutrophication. The eutrophication impact of the thermochemical process was about 33% lower than that of the bacterial retting process. For the rest of the environmental impact categories, the current bacterial retting process was approximately 20–25% lower than that from thermochemical retting. Bacterial retting even resulted in a negative ozone depletion potential, which represents the beneficial effect on the ozone layer. 

**Figure 3:**
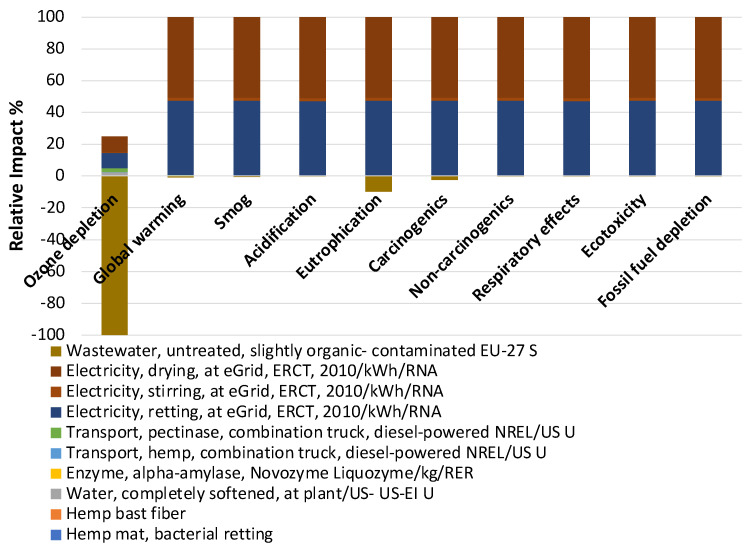
Material and energy contributions to the total environmental impacts of bacterial retting.

**Figure 4:**
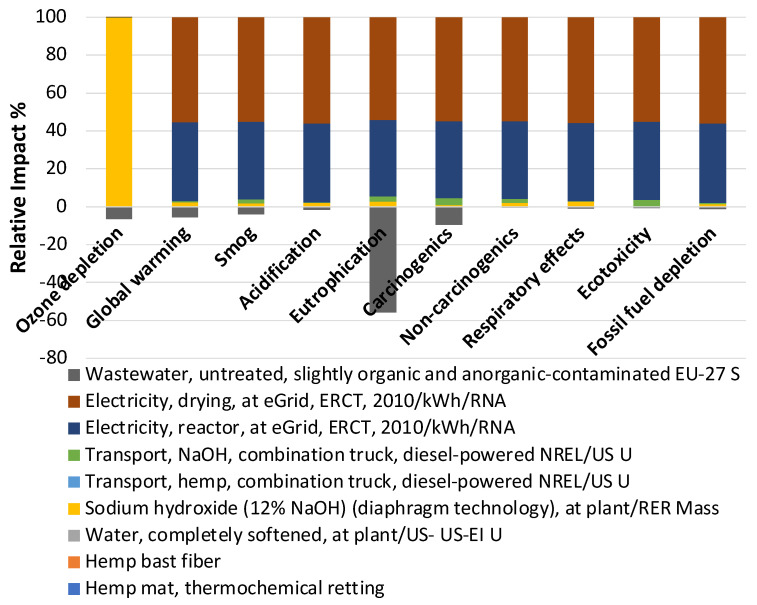
Material and energy contributions to the total environmental impacts of thermochemical retting.

The negative impact of bacterial retting processes on eutrophication is greater than that of thermochemical retting processes, mainly because the wastewater generated by thermochemical retting has a greater beneficial impact (−56%) on eutrophication (Figure 3 and Figure 4). Eutrophication is mainly due to the excessive accumulation of nutrients such as nitrogen and phosphorous in water [27]. It should be noted that nitrogen and phosphorous are critical to the soil quality for plant growth. A positive linear response of the hemp biomass yield to nitrogen fertilization was observed with the application of nitrogen fertilizer in industrial hemp cultivation [28]. Water used in bacterial retting was recyclable because the bacteria proliferated in water during the retting, which can be used to ret the next batch of fibers. The recycled bacterial liquid could also improve the concrete’s healing performance. Shaaban et al. incorporated Bacillus Subtilis into the concrete mix, and a significant increase in the compressive and tensile strengths of bacterial concrete was observed [29]. The thermochemical retting process generated much more unrecyclable wastewater than the bacterial retting process. Therefore, the impact of wastewater in thermochemical retting was much higher than in bacterial retting. Meanwhile, the beneficial effect of ozone depletion from wastewater can offset ozone depletion damage from the use of electricity and transportation. Compared to the thermochemical retting process, bacterial retting can reduce ozone depletion overall. Electricity consumption in both retting methods is the main contributor to the environmental impact. In addition to ozone depletion and eutrophication, the environmental impact is mainly due to the electricity consumed by drying for both methods. The environmental impact caused using electricity can be replaced using clean energy. For instance, the electricity used to provide a warm environment (40 °C) suitable for bacterial multiplication in the bacterial retting process can be reduced by using insulation materials or by using solar thermal storage systems. Compared to bacterial retting, thermochemical retting has a short retting cycle. However, 99.6% of the ozone depletion came from the use of sodium hydroxide (Figure 4). Despite the fact that the self-cultivated bacterial retting process has advantages over the thermochemical retting process in terms of environmental impacts in this lab-scale LCA analysis, uncertainties exist that need to be taken into account in real-world applications. Since currently little information is available about the LCI of pectinase, a well-developed alpha-amylase in the developed database was used in the analysis.

**Figure 5:**
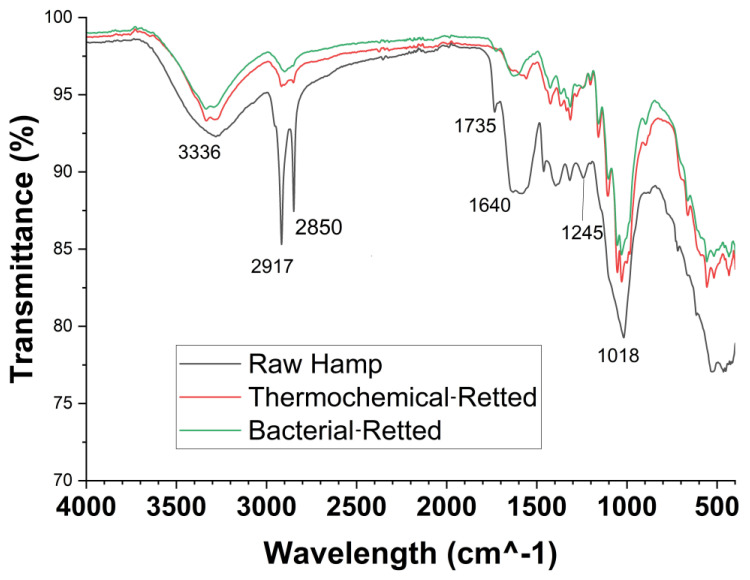
FT-IR spectra of raw hemp fiber, bacterial-retted fiber, and thermochemical-retted fiber.

The results of the FT-IR spectra of bacterial-retted fiber and thermochemical-retted fiber are shown in Figure 5, from which the common bands in 3100–3600 cm^−1^ are for OH stretching and 2800–3000 cm^−1^ are for C-H stretching. For the thermochemical-retted hemp fiber, the absence of the 1735 cm^−1^ (C=O stretching) peak could be due to the removal of carboxylic acids, aldehydes, or esters [30] from the fibers by sodium hydroxide. It is known that alkali is commonly used to extract hemicellulose and eliminate lignin from the plant-based material [31]. The spectral band around 1735 cm^−1^ was used to identify the pectin [32]. The peak at 1640 cm^−1^ (OH stretching) was due to the absorbed water in the tested samples [33]. Both retted hemp samples did not show the significant peak at 1245 cm^−1^ which was attributed to the C-O stretching in the hemicellulose or pectin [30], indicating that both retting procedures removed part of either the pectin or hemicellulose. The peaks between 995 and 1048 cm indicated the C-C, C-OH, and C-H rings and side-group vibration in the cellulose or hemicellulose [34].

## 4. Conclusions

From the gate-to-gate LCA models developed from this study based on data collected from the lab scale study, the enzyme-triggered self-cultured bacterial retting process had approximately 20–25% lower environmental impacts relating to global warming, smog, acidification, carcinogenics, non-carcinogenics, respiratory effects, ecotoxicity, and fossil fuel depletion than that from the thermochemical retting process. The major source of environmental impact for bacterial retting is the consumption of electricity. In terms of the ozone depletion impact, the enzyme-triggered self-cultured bacterial retting process caused almost no effect overall. The FTIR results indicate that hemp fibers obtained by bacterial retting showed comparable chemical characteristics to the thermochemical-retted fiber. This demonstrates the feasibility of using a self-cultured bacterial retting process to convert hemp bast into lignocellulosic fibers for further applications, such as reinforcement in composites. A future economic analysis comparing the different retting processes would be needed to add more insights into the different natural fiber retting processes.

## Figures and Tables

**Table 1:** Materials and energy inputs of the two processes to produce 1 g of retted hemp fiber.

Input				
Materials		Bacterial Retting	Chemical Retting	Unit
Raw hemp fiber		1.25	1.82	g
Enzyme		0.63	--	g
Sodium hydroxide		--	4.55	g
Water		125.00	272.73	mL
Electricity	Retting	0.09	0.11	kWh
	Stirring	0.0038	--	kWh
	Drying	0.101	0.147	kWh
Transport	Hemp	56.25	81.82	gkm
	Sodium hydroxide	--	11,818.18	gkm
	Enzyme	1187.5	--	gkm
Waste flow				
Wastewater		31.25	272.73	mL

**Table 2:** Cumulative energy demand of the two processes to produce 1 g of retted dry hemp.

Impact Category	Unit	Thermochemical Retting	Bacterial Retting
Non-renewable, fossil	MJ	2.82	2.18
Non-renewable, nuclear	MJ	3.61 × 10^−2^	−2.19 × 10^−4^
Non-renewable, biomass	MJ	9.57 × 10^−12^	2.08 × 10^−12^
Renewable, biomass	MJ	1.38 × 10^−5^	6.53 × 10^−7^
Renewable, wind, solar, geothermal	MJ	2.00 × 10^−4^	5.70 × 10^−7^
Renewable, water	MJ	4.51 × 10^−3^	−1.70 × 10^−5^
Total non-renewable	MJ	2.86	2.18
Total renewable	MJ	4.72 × 10^−3^	−1.58 × 10^−5^
Total energy	MJ	2.862	2.179

**Table 3:** Comparison of life cycle environmental impacts for bacterial retting process and thermochemical retting process.

Impact Category	Unit	Bacterial Retting	Thermochemical Retting
Ozone depletion	kg CFC-11 eq	−5.59 × 10^−12^	9.19 × 10^−10^
Global warming	kg CO_2_ eq	1.32 × 10^−1^	1.67 × 10^−1^
Smog	kg O_3_ eq	6.03 × 10^−3^	7.81 × 10^−3^
Acidification	kg SO_2_ eq	1.18 × 10^−3^	1.54 × 10^−3^
Eutrophication	kg N eq	1.27 × 10^−5^	8.49 × 10^−6^
Carcinogenics	CTUh	3.36 × 10^−10^	4.20 × 10^−10^
Non-carcinogenics	CTUh	5.36 × 10^−9^	7.19 × 10^−9^
Respiratory effects	kg PM2.5 eq	5.99 × 10^−5^	7.90 × 10^−5^
Ecotoxicity	CTUe	9.93 × 10^−2^	1.32 × 10^−1^
Fossil fuel depletion	MJ surplus	1.92 × 10^−1^	2.50 × 10^−1^

## Data Availability

The original contributions presented in the study are included in the article, further inquiries can be directed to the corresponding author.
